# Bedside Nurses' Perceptions of Effective Nurse-Physician Communication in General Medical Units: A Qualitative Study

**DOI:** 10.7759/cureus.25304

**Published:** 2022-05-24

**Authors:** Hirotaka Kato, Jessica M Clouser, Preetham Talari, Nikita L Vundi, Akosua K Adu, Kishore Karri, Kathy B Isaacs, Mark V Williams, Romil Chadha, Jing Li

**Affiliations:** 1 Hospital Medicine, University of Kentucky College of Medicine, Lexington, USA; 2 Health Services Research, University of Kentucky College of Medicine, Lexington, USA; 3 College of Nursing, University of Kentucky, Lexington, USA; 4 Hospital Medicine, Washington University School of Medicine, St. Louis, USA; 5 Internal Medicine, Washington University School of Medicine, St. Louis, USA

**Keywords:** content analysis, qualitative study, interprofessional collaboration, npc, nurse-physician communication

## Abstract

Background

There is a dearth of research on successful interventions to improve nurse-physician communication (NPC). An important step is identifying what matters to bedside nurses and their perceptions of effective NPC communications and actions.

Methods

We conducted three focus groups with a total of 19 medical unit nurses across two hospitals in one academic medical center in the United States. Using a convenience sampling strategy, five to eight nurses voluntarily participated in each focus group. The recording was transcribed verbatim and two independent coders performed coding and resolved any discrepancies in codes. Qualitative content analysis was pursued to identify themes and associated quotes.

Results

The presence of direct communication between physicians and nurses was identified as the first theme and perceived by nurses as very important. Additional themes related to physician communication and attributes emerged including collegiality and respect (e.g., engaging nurses as partners in patient care), attentiveness and responsiveness (e.g., listening carefully and addressing concerns), and directness and support (e.g., backing nurses up in difficult situations). Effective NPC is further facilitated by organizational structure, relationship development separate from patient care, and consistent/timely use of technology.

Conclusions

Hospital bedside nurses provided valuable insight into improved physician communication and what attributes contribute to more effective NPC. Most importantly, they emphasized the significance of physicians in supporting them with difficult patients.

## Introduction

Nurse-physician communication (NPC) is essential in the care of hospitalized patients. Ineffective NPC is associated with higher mortality and adverse event rates [1-4]. Importantly, the Institute of Medicine, now the National Academy of Medicine, holds healthcare organizations accountable for adverse events originating from poor NPC [2,5]. In the past two decades, results from an increasing number of studies aiming to understand and improve NPC have been published. A review of previous studies identified four main themes from studies of NPC: 1) differences in communication styles, 2) facilitators, 3) barriers, and 4) interventions to improve NPC [6]. Studies attempting to improve NPC targeted different communication styles and tried to mitigate barriers to NPC but have shown mixed results. One common approach is to provide communication tools to standardize and improve information exchange such as a daily goals worksheet [7] and the adoption of the Situation-Background-Assessment-Recommendation (SBAR) framework [8,9]. Another compelling approach is to restructure patient care workflow to facilitate face-to-face communications [10-14]. For instance, O’Leary et al. documented enhanced NPC and agreement on the plan of care through localizing internal medicine physicians to particular units and structured interdisciplinary rounds [12,13]. Li et al. implemented the interprofessional team innovation model, which added to geographic localization by having the bedside nurse, a case manager, pharmacist, and physician round together as a team [11]. This approach improved NPC perceptions and reduced 30-day readmission rates with no increase in costs.

Even though NPC has been extensively studied, a significant gap still exists in how these various interventions influence nursing and physician competency in interprofessional communication [15]. Notably, past studies measured NPC in many ways. The most common approaches included surveying participants about their perceived effectiveness of NPC, quality of information exchange, and frequency of communications. Moreover, a variety of instruments exist that measure NPC, interprofessional communication/collaboration, and physician non-technical skills. They are, however, mostly self-assessment, perceptions, or team performance, not suitable to be used as a workplace-based assessment for feedback and improvement [16-19].

Despite acknowledgment by the National Academy of Medicine and medical and nursing education accreditation bodies more than 20 years ago that healthcare professionals need to develop a shared culture of collaborative teamwork and communication [20], little progress has been made. The lack of practical and validated tools that can be used by bedside providers to assess their interprofessional communication skills, including NPC, represents a major barrier. As past studies have consistently shown, nurses and physicians have different perspectives, roles, and workflows in patient care [6]. To better understand these aspects of NPC, we undertook a qualitative study to explore how bedside nurses working in general medical units view effective NPC and what facilitates NPC.

## Materials and methods

Study setting and participants

From a constructivist perspective, we conducted focus groups at two hospital facilities within one academic medical center system, University of Kentucky, Lexington, Kentucky, United States: University of Kentucky Albert B. Chandler Hospital, a quaternary referral hospital, and University of Kentucky Good Samaritan Hospital, a community hospital. Nurses working in any of 10 general medical units at these two hospitals were eligible to participate. There were 10-36 beds on each unit. The nurse-to-patient ratio ranged from 3:1 to 5:1 depending on the acuity of the patients. All medical patients were managed by hospitalists on direct care and/or teaching teams. These teams had different ranges of geographic cohorting (up to 90%). No exclusion criteria were applied based on age, gender, ethnicity, experience, or length of employment since no demographic information was collected. Our research team consisted of hospitalists, nurses, and health services researchers to ensure a range of perspectives in the study planning and interpretation. 

Ethics

All participants provided written informed consent before participating in the focus group interviews. The study protocol was approved by the Institutional Review Board at the University of Kentucky (approval number 49608).

Sampling

We used a convenience sampling approach to recruit nurses for the focus groups. Nurses in the selected general medical units were the suitable population for our study because they had plenty of opportunities to interact with physicians caring for medical patients. We intended to include any nurse in these units irrespective of their background such as length of employment. The nurse managers in these selected units notified their nurses of the opportunity to participate via a group email. Focus groups were held during the lunch hour with food provided. Participation of nurses was voluntary, and individuals’ attendance was not communicated to nurse managers nor to clinical members of the research team to ensure anonymity. Three focus groups were planned, consistent with prior research that found 80% data saturation from a range of two to three focus groups and 90% data saturation from a range of three to six focus groups [21].

Data collection

We chose the focus group format to allow for participants to respond to one another’s comments and openly discuss potential differences and similarities in experience or preference. Each of the three focus groups included five to eight participants with a total of 19 participants. Due to the co-located nature of participants and research team in the same health system and the sensitive nature of the subject matter, we did not collect demographic data from participants to preserve their confidentiality from the physician members of the research team. In addition, no physicians or nurse supervisors were present during the focus groups. Instead, one of three non-clinician researchers (JMC, NLV, AKA) with experience and training in qualitative research techniques facilitated each focus group, with a second serving as a note-taker. Interviews began with a broad general question asking how participants define effective, collaborative communication; subsequently, using open-ended questions to elicit information with minimal bias (Table 1). The focus groups were audio-recorded with prior written consent from all participants. To maintain confidentiality, individuals’ names were not used or recorded at any stage of the data collection process.

**Table 1:** Semi-structured interview guide

Semi-structured interview guide	
1)	What does effective communication mean to you and what specifically would it look like?
2)	Please share your experience communicating with physicians
a)	How would you like physicians to communicate with you?
b)	What traits in physicians make you feel like they are communicating well?
3)	Can you describe a specific situation with a physician that you would consider to be an example of effective collaborative communication? What did the physician do?
4)	Can you describe a time when you felt that communication with a physician was ineffective?
5)	Is there anything that you want to share with me about communicating with physicians that I have not already asked?

Data analysis

Each audio file was transcribed verbatim and quality checked by a second researcher to ensure accuracy. Final transcriptions were imported into an NVIVO 12 software (QSR International, Doncaster, Australia) workbook. We employed an inductive approach, using qualitative content analysis [22] to allow codes and relationships among them to emerge from the data.

The first focus group transcript was used to develop the initial coding structure using open coding. Two independent, non-clinician investigators with experience in qualitative research (JMC and NLV) independently coded this transcript by reading the data line by line, to identify underlying meaning or concepts for initial code identification, documenting their observations about properties of and possible relationships between codes through analytic memos. After the initial round of coding was complete, all three members of the analytic team (JMC, NLV, AKA) met to discuss emerging codes to develop an initial codebook including emerging themes and categories. Discrepancies were resolved through discussion and reference to the original text until consensus was reached. The coding template was then used to code the remaining transcripts by two independent coders. After the independent coding of each transcript, the analytic team met to discuss consistency in the application and interpretation of existing codes as well as the possible emergence of new codes, ideas, or themes and the relationships among them. By the third and final focus group transcript, no new concepts or codes emerged, suggesting that data saturation was achieved [21].

The analytic team shared emerging themes with the research team including physicians and nurses along with thick or detailed descriptions of the codes and associated text to aid in coding validation and interpretation. They repeatedly provided feedback on how themes were categorized and/or phrased. As a result of this iterative, collaborative process, dominant themes related to nurses’ characterizations of effective NPC were finalized by group consensus. These dominant themes were expressed in two or three focus groups, often by multiple participants.

## Results

Part I. Nurse characterization of effective physician-nurse communication

Direct Communication

Effective communication was often characterized by the participants as simply the presence of direct communication between nurses and physicians. They stressed the importance of knowing what is going on in real-time to provide the best possible care: “*I need to know everything that’s going on in the plan of care…That’s effective to me. Just let me know what’s going on*." However, too often, according to nurses, the patient served as a conduit in communications between nurses and physicians regarding the care plan, which could lead to confusion on behalf of the patient and the care team.

There are a lot of people here who have a very low literacy rate of health. So, you know there can be times whereby not taking the nurse with you (physician) … patients will tell us stuff and it’s like, ‘That sounds like a horrible idea. Like you’ve got a broken leg and you are telling me the doctor says he wants you to walk?’

The inclusion of the nurse during the physician rounds was emphasized throughout each focus group multiple times, highlighting the specific importance of direct, real-time communication that not only included the nurse and physician, but also the patient. Participants noted that physician/nurse co-rounding helped ensure patients understood the plan of care. Participants cited multiple reasons why the nurse’s presence may enhance communication between the physician and the patient, including power differentials that may make patients feel intimidated to ask questions, differences in health literacy, or accent differences between patients and physicians that could impede understanding. Most importantly nurse saw their roles as patient advocates.

It’s really important for us to be there at the bedside with the physician because patients will smile and nod at the doctor and not ask any questions and be like “yes I understand” because they are intimidated … And then when the doctor leaves, they’ll call me in there and ask me a bunch of questions and “well I wasn’t in there, I don’t know”

Some of our physicians have thick accents like the doctor will have a whole conversation and they’ll be like "okay" and (the physician will) leave the room and … (the patient will) be like “what did he say?” … and it’s really helpful for us to be there even if they don’t have an accent regardless. It may sound like you’re speaking another language because they’re using medical lingo and the patient just has no idea what (the physician) was saying.

Collegiality and Respect

The quality most frequently described by participants as important to NPC was collegiality and respect, i.e., engaging nurses as partners in patient care and respecting their role on the care team. Nurses wanted to be respected for their training, experience, and familiarity with the patient. They wanted physicians to ask for their input and to share their rationale behind decisions to help achieve improved patient care.

I think that when residents or physicians value nurses as part of their team and like we’re their partner in taking care of the patient and they appreciate and respect the nurse, and that’s really shown in how they talk to us, or how receptive they are to our suggestions or listening to us… it’s more of a peer relationship versus like “I know everything and you don’t know anything and I’m not listening to you cause I’m the boss.”

I think part of it comes down to culture and respect. So, if you have that culture ingrained the first thing you do when you pre-round is you go to your patient and go find your nurse …. But that culture of getting into that habit and the respect of realizing that it’s a nurse-physician partnership in care and not "I’m the doctor and you do what I say. You follow my orders," but we are collaborating, truly, in care.

Focus group participants identified physicians as effective communicators when they take a team approach or when they try to problem-solve with nurses together.

We have some physicians that are phenomenal, and they will come to you, and they will say “Hey Nurse Red, I’m going to go see you know room XXX, will you go in there with me? He’s been wild today cussing and yelling and I’m going to talk to him about that and I would like to have you with me.”

I always have more respect for the residents and even the attendings who say ‘You know what? I’m not sure how to do this. Do you know?" or "Let’s figure it out." And they’ll sit down next to you…

Attentiveness and Responsiveness

Participants talked about wanting to feel that physicians were paying attention to them, making eye contact, taking time to address their concerns or gather their input, and providing responses in a timely manner.

Feeling like they’re really paying attention to what you’re trying to tell them and that they’re like taking the time to address your concerns. Because sometimes they can just blow you off. 

 (Effective communication is) when physicians, like, when they are available when you need them, and they answer their pages in a timely manner.

Conversely, participants expressed frustration when a lack of follow-through occurred.

Another thing I find frustrating though is… when you ask the doctor for something, whether it’s face-to-face or through (Health Insurance Portability and Accountability Act (HIPPA)-compliant texting software also known as clinical communication and collaboration platforms (CC&C)) and then they don’t do it. They’ll tell you they’re going to put the order in or take the order out and then it just never happens. There’s some sort of breakdown there.

Directness, Consistency, and Support

Participants reported that physicians’ directness and clarity with nurses and patients, buttressed by consistent follow-through, also characterized effective communication. Nurses repeatedly highlighted this as important in nurse-physician communication, and noted its importance with patients, especially with more complex and non-adherent patients. When messages coming from the physician and nurse were inconsistent with one another and patients’ understanding did not reflect the reality of the situation, a nurse’s authority and rapport with the patient could be undermined.

You know, this morning during rounds they said, we’re not giving you pain medicine (i.e., opiates). So we’re telling them no, have some Tylenol, here’s a hot pack. And then it’s like they (patient) finally like throw a big enough toddler-level tantrum to where the physician will come and be like, “I’m so sorry. Here you go. Here’s, here’s what you want. I know that you know, your nurses just fought you for eight hours over this, but we want you to stay."

Alternatively, such tension and conflict could be avoided when physicians were direct with patients and expressed support for the nursing staff, because as one nurse noted, “we’re the ones who are having to deal with this during the day.”

(The physician) introduces himself and me, and then he tells the patient what we allow and what we don’t allow. And he’s very matter-of-fact about it and he’s very supportive of the staff on the floor, and if the patient breaks that contract, then he does what he says he will do. 

Part II. Drivers of effective communication from nurse perspectives

Participants discussed effective communication as multifaceted. They identified key inputs that facilitate effective communication, that, when used, drive the "operation" of communication. These drivers are deemed essential.

Driver 1. Organizational Structure

Routine, Interdisciplinary Rounds: Some participants recruited to the study worked in units that had adopted daily interdisciplinary rounds, whereby hospitalists, nurses, pharmacists, and case managers routinely conducted bedside rounds. Nurses from these floors expressed satisfaction with the degree of access to face-to-face communication with the physicians and patients; more so than nurses recruited from units without this practice.

…how we round, Monday through Friday, with the physician, the pharmacist, the nurse, and the case manager, that actually opened up a whole new world for nurses and physicians…where we’re rounding on each patient every single day has allowed the physician to see, not just what we’re doing for the patient, but what we’re doing to help (the physician) as well… they realize that we’re a team.

Co-Located Offices: Participants also noted that access to face-to-face communication was enhanced when physician offices were located near their patient units. Participants working in units with quick access to face-to-face communication with physicians had fewer complaints about wait times for returned calls/messages and expressed more positivity about collaboration on patient care and access to physicians.

The good thing about (certain floors), our physicians are on the floor, and they sit just right around the corner. So if we have any questions, we can kind of peek our head in to whatever office that they’re in.

Driver 2. Building Relationships and Rapport

Participants described physicians who build a personal relationship or professional rapport with nursing staff made it easier for nurses to engage them in important conversations about patient care. Examples of how professional rapport could be established included: checking in with nursing staff, asking for their opinion or their experience with their patients, or co-rounding. Participants also described how asking about their family and life outside of work helped build the relationship and their comfort level with each other.

Having a good rapport with all the physicians, I believe, it’s a great way to have effective communication. Being able to even just talk outside of even something going on with the patient. I believe all of our physicians have been here for quite some time that, you know, they are able to sit down with us and we are able to work as a team to kind of figure out what we need to do for the patient to make their stay more efficient, their health, and just their safety and everything, the top priority.

Driver 3. Consistent and Timely Use of Technology for Communication

Notes in the electronic medical record and secure messages via pagers or the CC&C were all described by the participants as tools used among nurses and physicians to exchange information in real-time.

(CC&C) is so much more convenient to be able to text. And if it’s something you need to actually have a phone conversation, then you can call them or…you can ask them to call you. It’s just more efficient.

For the tools to be effective, however, their use needed to be consistent and timely across service lines. In some instances, participants noted a lack of follow-up with no action taken despite communicated commitment.

Driver 4. Organizational Culture

Although technology was acknowledged as a valuable communication tool, participants commented that it was no substitute for a culture of communication; “*no matter what kind of tools you have, if you don’t have that priority of communicating, you’re not going to use it.*” Such organizational culture was repeatedly referenced as influencing individual physicians' approaches to communication. Having a team-centered approach to care, in which nurses, physicians, and other care providers worked together as a team, was frequently discussed as a facilitator to effective communication. One participant commented that effective communication occurred because the physicians they worked most closely with, the hospitalists, saw them as a team:

I think that is, they realize that we're a team. Not that I'm saying that they didn't realize it before, but I think that is the bigger picture. . . is the teamwork involved in that. And I feel like that’s how our relationship with our physicians (i.e., hospitalists) are completely different from the ones that are our specialty teams. 

One part of the culture of a team-based approach to care was evidenced in physicians keeping both the nurse and the patient informed regarding updates in the care plan and the rationale behind those decisions.

If you change orders and stuff … It would be nice if they explained to (the patient) what their plan was and then come to us at times and say, ‘Hey I’ve explained to the patients we’re not going to be giving this IV pain medicine anymore; we’re going to be trying the pain pills." That way it’s not a shock to the nurse and it’s not a shock to the patient. 

Just as having a team-based culture of patient care facilitates effective communication, participants described how power differentials and role expectations could hinder both nurses and physicians from feeling comfortable asking questions of one another. A culture of teamwork was discussed as particularly relevant in the context of a teaching environment in which relatively new residents work alongside nurses with longer tenure and more experience. Participants reported that residents might not realize the value that nurses bring to patient care.

A lot of times as nurses we may have more experience on the floor than say a first-year resident in August. But also, we’re more familiar with the (hospital’s) way of doing things and assistive care… And I don’t know if within the teaching teams if there’s this culture of “if you have to ask for help, you don’t know what you’re doing therefore you’re incompetent as a provider,” but it’s always to the detriment of the patient.

I think that you get a lot of younger physicians who don’t really understand the knowledge base that nurses have, and they don’t value that, and they don’t listen, and they demean it.

Similarly, the culture and expectations set by the attendings influenced how and how much residents communicated with nurses.

I really like that resident, but you know this month it’s going to be rough because they’re with that attending so they’re probably not going to communicate with you as well, they’re not going to let you know when they are rounding.

We also identified the group of themes that represents the perceived benefits of effective NPC and summarized them in Table 2 with representative quotes.

**Table 2:** Perceived benefits of effective nurse-physician communication (NPC)

Effective NPC…	Representative quotes
Builds skills and knowledge base of the care team	“…it’s a partnership, so teach us. And then I can also make sure that the patient understands why we are doing these things. And just coming to the table, like, with mutual respect and with the air of wanting to listen to each other.” “If the nurse isn’t understanding why you want to do something, or they are questioning you, use that as an opportunity to educate them. Like, I don’t know everything. I want to know! I want to understand why you want to order this.”
Saves time	“…get the nurse, you know, have the nurse in the room with you. Because (the nurse is) a tool for the doctor and an advocate for the patient. And when there’s the three of you in the room, you’ve eliminated so many questions and saved so much time.”
Improves patient care and experiences	“If we don’t know as nurses what you want us to do, we can’t do it. So you’re going to be unhappy as the provider, the patient is going to be unhappy.”

## Discussion

Our qualitative study sought to understand what matters most in nurse-physician communication from the perspective of hospital nurses’ working in general medical units with the goal of eventually developing a communication assessment tool. Though facilitators and barriers to NPC have been extensively studied, we explored this aiming to identify what is most important for effective NPC. This information should be useful to develop functional assessment tools that can be utilized in intervention efforts to improve NPC. We believe that unit nurses are in the best position to assess physicians’ performance on NPC since collaboration between nurses and physicians makes up the largest workforce segment in care [23]. Nurses highlighted how direct communication either face-to-face, via text, or on the phone must be present. Collegial, responsive, and supportive physician communications enable effective NPC. In addition, effective NPC is facilitated by developing friendly relationships that include communication about non-work activities, effective technology, and an organizational culture that emphasizes teamwork. This delineation of effective NPC approaches will be a start toward measuring and improving NPC in a consistent manner.

Significant commonalities were found between our findings and past studies. Our main themes - the presence of direct communication, collegiality and respect, attentiveness and responsiveness, consistency, and support - include the notions of trust, collaborative attitudes, teamwork, and lack of communication opportunities reported by similar studies [24,25]. Partnership and relationship between nurses and physicians have been repeatedly found as key facilitators of NPC [26,27]. Language and cultural barriers reported by Robinson et al. [25] did not strongly emerge in our study between nurses and physicians specifically, although physician-patient language barriers did emerge. Tan et al. summarized these reported themes of NPC, including common understanding, trust and respect, collaborative attitudes, lack of communication opportunities, modes of communication, objectionable communications, insufficient information, selective communication, language, and culture [6]. But they did not provide specific guidance on assessment or which themes were most important. In developing an instrument to measure health professionals’ NPC skills, these reported themes can be difficult to transform into assessment items. An item like, “did this physician show collaborative attitudes in NPC?”, asks evaluators to generate an accurate summative assessment, but doing so typically requires a significant number of observations. A single negative interaction may overwhelmingly influence the evaluation while not providing actionable feedback.

**Figure 1:**
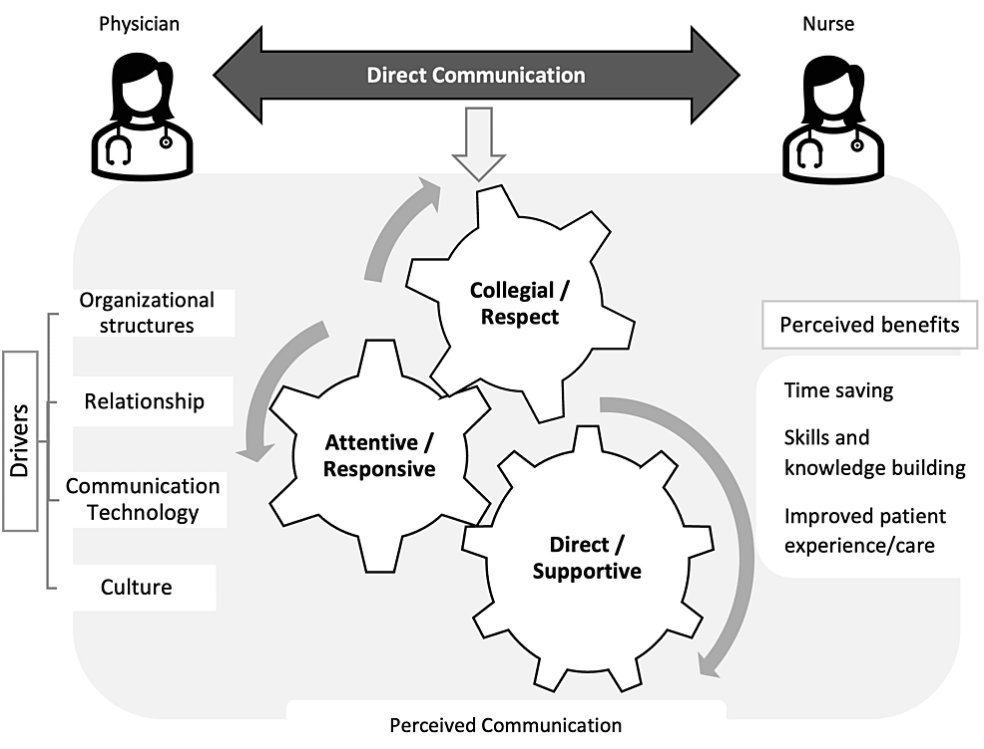
Visual illustration of identified themes (communications, drivers, and benefits) of effective communication from nursing’s perspectives in inpatient medical units

Our study results help prioritize what is most important in measuring NPC. Figure 1 illustrates the interrelationships among our own themes based on physicians’ communication and attributes described by the nurses. These themes and their quotes can be used to devise communication-based assessment items to measure physicians’ NPC skills. For example, physicians with high NPC skills would introduce themselves to their patients’ nurses during rounds, seek input from nurses, value their opinions, and invite them to the bedside when seeing a patient. They would demonstrate a team-based approach to problem-solving, rather than simply giving orders to nurses. Power differential issues can impede effective NPC [28] but the above approaches can overcome such issues by working toward the same patient care goals. Our model of effective NPC (Figure 1) can also be used as a guide to educate physicians and residents on effective communication. There is an urgent need to educate providers on NPC skills given a collegial nurse-physician relationship is associated with better safety and quality outcomes [29]. Furthermore, nurses’ perceptions toward NPC are affected largely by the presence of effective communication, perceived respect, and collaborative attitudes [30].

Our study findings should be interpreted with caution. First, this was a single-center study even though our themes significantly overlap with previous study findings. The study site as an academic medical center is also a limitation in generalizability, given the extensive use of trainees and a more complex and sicker patient population. Second, we did not collect our participants’ demographic data to ensure confidentiality. This limits our interpretations of the reason why the cultural and language barrier between physicians and nurses [25] did not emerge in our study. Last, we conducted only three focus groups with a total of 19 nurse participants. Nonetheless, we appeared to reach data saturation by the third focus group and results were consistent with prior studies of NPC and research that similarly showed high levels of data saturation after three focus groups.

Even with these limitations, our findings build on prior NPC studies and provide new data to inform a future survey instrument for nurses to assess physician communication with nurses. Importantly, our study findings highlight and confirm emerging difficulties that nurses face related to verbal and physical abuse from patients and the importance of physicians in mitigating this danger through consistent and supportive communication. We also identified what factors are most important to nurses in communication between nurses and physicians. The results of the study can help provide a scientific basis for hospital-based assessment of NPC.

## Conclusions

Previous studies successfully encompassed factors involved in NPC. However, bedside nurses and physicians often struggle to find a better way to communicate due to increasing time pressure on both. This study finding illustrates and breaks down the dynamics of effective NPC into three major components: (1) the occurrence of direct communication as the fundamental step, (2) the physician's approach to communication, and (3) organizational-level factors such as structure, culture, and communication technology. This allows bedside clinicians not only to assess their current practice and gaps but also to start observing and improving their performance of NPC in the medical units. Future studies should develop a practical and validated instrument to measure NPC competency/performance based on physicians’ communication approaches that matter most to the nurses.
